# CRISPR interference in a *Streptococcus agalactiae* multi-locus sequence type 17 strain

**DOI:** 10.1128/jb.00376-25

**Published:** 2026-01-14

**Authors:** William D. Cutts, Aidan W. Flanagan, Brice K. Gorman, Audrey Sweten, Bryan J. Estrada, Vishwas N. Subash, Benjamin T. Klemp, Kathryn N. Seely, Austin D. Sandobal, Katelin R. Stilen, Taksh Vaghela, Areebah Mehvish, Jacob F. Wood, Alexus M. Govert, Kristin E. Hobson, Gideon H. Hillebrand, Thomas A. Hooven, Brandon J. Kim

**Affiliations:** 1 Department of Molecular and Cell Biology, University of Texas at Dallas, Dallas, Texas, USA; 2 Department of Biological Sciences, University of Alabama 164491, Tuscaloosa, Alabama, USA; 3 School of Animal and Comparative Biomedical Sciences, University of Arizona 737151, Tucson, Arizona, USA; 4 University of Texas at Austin 12330, Austin, Texas, USA; 5 Department of Neurosciences and Psychiatry, University of Toledo College of Medicine and Life Sciences 89021, Toledo, Ohio, USA; 6 Belmont Abbey College 1633, Belmont, North Carolina, USA; 7 Department of Pediatrics, University of Pittsburgh School of Medicine 12317 , Pittsburgh, Pennsylvania, USA; 8 Richard King Mellon Institute for Pediatric Research, University of Pittsburgh Medical Center 6595, Pittsburgh, Pennsylvania, USA; 9 UPMC Children’s Hospital of Pittsburgh, Pittsburgh, Pennsylvania, USA; 10 Department of Microbiology, Heersink School of Medicine, University of Alabama at Birmingham 318277, Birmingham, Alabama, USA; Indian Institute of Technology Bombay, Mumbai, Maharashtra, India

**Keywords:** CRISPR, Group B *Streptococcus*

## Abstract

Group B Streptococcus (GBS), a common colonizer of the human genital and gastrointestinal tracts, is a leading cause of neonatal bacterial meningitis, which can lead to severe neurological complications. The hypervirulent serotype III, sequence type 17 (ST-17) strain COH1 is strongly associated with late-onset disease due to its unique set of virulence factors. However, genetic manipulation of ST-17 strains remains challenging, limiting the ability to study key pathogenic genes. In this study, we developed a CRISPR interference (CRISPRi) system utilizing an endogenous catalytically inactivated Cas9 (dCas9) in the COH1 strain, enabling targeted and tunable gene expression knockdown. We confirmed the efficacy of this system through hemolysis assays, qPCR transcriptional analysis, and *in vitro* infection models using human brain endothelial cells. The CRISPRi system successfully produced phenotypic knockdowns of key virulence genes, including PI-2b, *srr2*, and *iagA*, reducing adhesion, invasion, and inflammatory responses at the blood-brain barrier (BBB). This platform enables rapid gene knockdowns for functional genomics in ST-17 GBS, enabling high-throughput screening and pathogenesis research.

**IMPORTANCE:**

Group B Streptococcus (GBS) remains the world's leading cause of neonatal meningitis. GBS-host interactions at the blood-brain barrier (BBB) are dependent on bacterial factors, including surface factors and two-component systems. Multi-locus sequence type 17 (ST-17) GBS strains are highly associated with neonatal meningitis, and these strains harbor many virulence factors for infection at the BBB. Historically, these factors have been studied using traditional knockout mutagenesis, which has been challenging in the most common ST-17 lab strain, COH1. This study utilizes CRISPR interference (CRISPRi) to generate rapid expression knockdown. This study validates a CRISPRi-enabled COH1 dCas9 strain as a versatile tool for probing GBS pathogenesis at the BBB.

## INTRODUCTION

Group B Streptococcus (GBS), also known as *Streptococcus agalactiae*, is a Gram-positive bacterium that asymptomatically colonizes the genital and gastrointestinal tracts of 20%–30% of healthy individuals (1, 2). However, during or shortly after birth, GBS can opportunistically infect neonates and infants, leading to conditions such as sepsis, pneumonia, or meningitis (3–6). Worldwide, GBS is the leading cause of neonatal bacterial meningitis, which is uniformly fatal without medical intervention (1, 2). While modern medical interventions have transformed GBS meningitis from a uniformly fatal illness to an often curable one, mortality remains at 5% to 10%, with survivors facing long-term neurological sequelae, such as blindness, deafness, seizure, and stroke (1, 7–9). Additionally, GBS can asymptomatically spread to other body sites and cause disease in adults, including soft tissue infections, bacteremia, urinary tract infections, endocarditis, and meningitis (3–5). Neonatal invasive GBS disease is classified into two types: early-onset disease, which occurs within the first 7 days of life, and late-onset disease (LOD), which develops between 7 and 90 days of life. Ninety-seven percent of neonatal invasive GBS diseases are caused by serotypes I–V, and serotype III accounts for 43% of early-onset disease and 73% of late-onset disease (1, 5, 8). LOD presentation is most commonly meningitis, but can also present as urinary tract, joint, bone, and soft tissue infection, as well as pneumonia and bacteremia (8, 10, 11).

Some GBS strains are more strongly associated with neonatal disease than others. Many strains that fall within serotype III sequence type 17 (ST-17), considered a hypervirulent sequence type, are significantly associated with neonatal meningitis and particularly LOD (5, 8). A significant contributing factor to ST-17’s hypervirulence is the number of adhesins and virulence factors shared across this clade. These include the serine-rich repeat proteins (Srr-1/2), GBS pilus tip adhesin PI-2b (homolog to *pilA* in GBS str. NCTC 10/84), hypervirulent GBS adhesin, laminin binding protein, streptococcal fibrinogen binding protein A, and Group B streptococcal surface protein C, as well as the two-component systems CovR/S and CiaR/S, which have all been described previously through traditional allelic-exchange mutagenesis or transposon mutagenesis (2, 12–24). One such ST-17 strain, COH1, is presently one of the most commonly utilized ST-17 strains in the laboratory for studying GBS meningitis (2, 13, 25–31). Given its clinical relevance and widespread use in pathogenesis research, there remains a strong need for more robust genetic tools to dissect virulence mechanisms in this strain, particularly at the blood-brain barrier (BBB). GBS utilizes an endogenous type II-A CRISPR-Cas9 system, much like *Streptococcus pyogenes*, and this system has recently been used to screen candidate genes using CRISPR interference (CRISPRi) (32). This process involves site-directed mutagenesis of key catalytic residues RuvC-like and HNH domains (D10 and H845, respectively), which yields the ability to produce targeted, tunable expression knockdown in any candidate gene containing the appropriate NGG protospacer adjacent motif (PAM) sequence (32, 33). Until now, CRISPRi has been utilized in serotype Ia and serotype V strains which are not as translatable to study at the BBB. We hypothesize that such a system in COH1 will provide a faster path to *in vitro* loss-of-function phenotypes, facilitating the speed of research on genes of interest. Traditionally, constructing allelic exchange mutants requires laborious screening and optimization. While CRISPRi is not a replacement for clean gene deletion, it enables the generation of transcriptional knockdowns in a much shorter timeline from design to experimentation. Therefore, CRISPRi enables the potential identification of a phenotype of interest before the generation of a traditional mutant. We report here the use of our hCMEC/D3 brain endothelial cell model to study infection, specifically quantification of bacterial adhesion, invasion, and chemokine expression after knocking down various virulence genes. Additionally, in the process of generating the COH1 dCas9 mutant for this paper, we report the first use of a Cas12a-based system in the generation of a markerless point mutation in GBS. These findings will be of use in facilitating GBS research.

## RESULTS

### Generation of *Streptococcus agalactiae* str. COH1 dCas9

**Fig 1:**
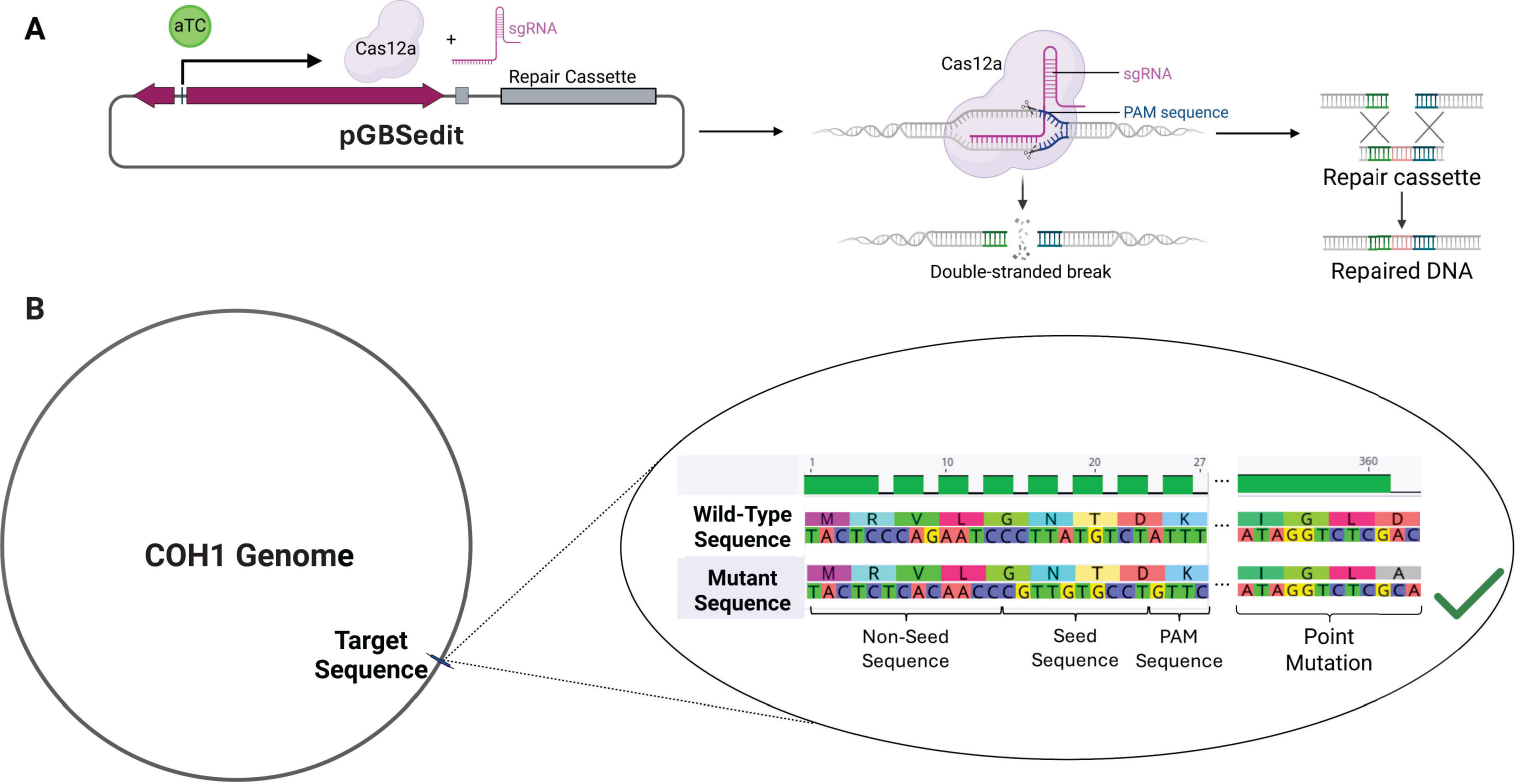
Cas12a-based point mutation for CRISPRi. (**A**) Graphical representation of GBS point mutation process, showing anhydrotetracycline induction of Cas12a and sgRNA expression on the pGBSedit plasmid. Also pictured is the inclusion of a repair cassette, containing the homology needed for repair and carrying the desired mutation. (**B**) Depiction of the genotypic changes needed to generate a point mutation, showing both a hypothetical point mutation (D360A), as well as the silent mutations in the sgRNA target site necessary to protect the plasmid and mutant strain from Cas12a cutting. These silent mutations must be designed on the plasmid repair cassette for the mutation process to be successful.

**Fig 2:**
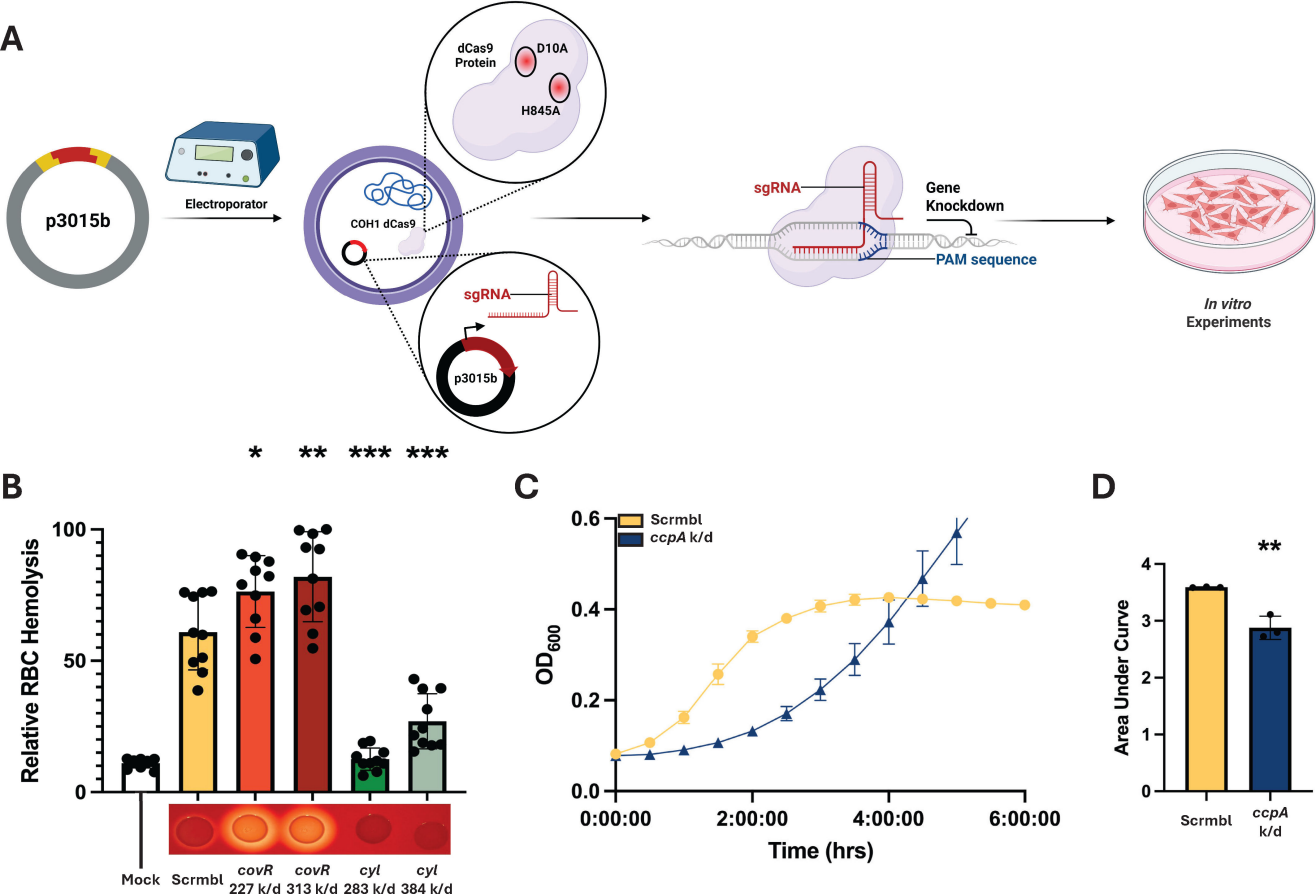
Verification of phenotype changes. (**A**) Graphical depiction of the knockdown generation process. sgRNAs are cloned into the shuttle vector p3015b, then electroporated into *Streptococcus agalactiae* str. COH1 dCas9. These transformants should now have a knockdown phenotype and can be used for subsequent *in vitro* experimentation. (**B**) Hemolysis assay data, with Mock being PBS-treated sheep’s blood, “scramble” (scrmbl) being a control infection utilizing a non-targeted sgRNA, as well as two variants of knockdowns of *covR* and the *cyl* operon. Beneath each bar graph is a visual representation of the hemolytic phenotype of each transformant using bacterial growth on sheep’s blood agar, with the *covR* knockdowns showing more hemolysis relative to the control and the *cyl* knockdowns showing less hemolysis. (**C**) Representitive technical triplicate growth kinetics curve showing lengthening of the lag phase when knocking down the essential gene *ccpA*. (**D**) Further quantification using the area under the curve (AUC) was performed, showing a significant AUC reduction with knockdown of *ccpA*. One-way ANOVA was performed, *P < 0.05, **P < 0.01 and ***P < 0.001. All experiments were performed in biological and technical triplicate (*n* = 9). For AUC analysis, triplicate values were calculated from kinetics curves and Student’s *t*-test was performed, **P < 0.01.

To utilize CRISPRi in GBS, we opted to make use of the endogenous GBS *cas9* gene, point-mutating it to generate a COH1 strain with catalytically deadened Cas9 (dCas), into which we transform a single plasmid expressing the single guide RNA (sgRNA) used to target dCas9 to the site of interest in the genome (Fig. 2A) (32, 34). The mutant was generated using two approaches, with the first point mutation inactivating the HNH-like domain (H845A) generated using classical allelic exchange methods, and the second point mutation inactivating the RuvC-like domain (D10A) generated with a Cas12a-based gene editing method (32, 33, 35, 36). The Cas12a mutagenesis process required targeting of the genome for cutting and presentation of a repair cassette on pGBSedit that carried our target (D10A) mutation, as well as silent mutations altering the sgRNA target site and PAM to protect any successfully edited mutants (Fig. 1A). For these silent mutations, we selected codons frequently used by GBS to avoid introducing rare codons that might alter gene expression (37). This is the first instance of this system being used to generate a markerless point mutation in GBS (Fig. 1A). Mutagenesis was confirmed via Oxford Nanopore whole-genome sequencing (Plasmidsaurus) to verify the correct genotype (Fig. 1B) (38). No growth kinetics changes were observed with this mutant (Fig. S1).

### Verification of knockdown phenotypes

To confirm that the mutant strain would show an altered phenotype when targeted with an sgRNA, we performed a hemolysis assay utilizing sgRNAs targeted to the *cyl* operon and the known virulence repressor *covR*, which regulates *cyl* expression (2). To verify reduced hemolytic activity, a 1% red blood cell (RBC) solution was aliquoted into a 96-well plate, infected, and then combined with a PBS suspension of a GBS + sgRNA transformant. Two guides were used for each target gene to evaluate the potential for modulation of the knockdown phenotype. Control groups were instead treated with an equal volume of PBS or with 0.1% Triton X-100, as well as an experimental control carrying a “scramble” sgRNA sequence that lacks a genomic target in COH1 and should therefore produce no change in phenotype. Triton-X is a detergent that will lyse the RBCs nearly completely, providing a maximum lysis level to normalize data. After a 1-h incubation, cell debris was pelleted, and supernatant was transferred to a new 96-well plate, and absorbance was read at 415 nm to read hemoglobin release as a proxy for the degree of RBC lysis. Results were normalized to the reads of Triton-X-treated samples. Almost all the GBS-infected wells showed some degree of lysis compared to PBS mock wells. The strains harboring *cyl*-targeted guides showed reduced hemolysis relative to the scramble control, and the *covR*-targeted strains showed increased lysis, demonstrating that the guides and CRISPRi system produce expected phenotypes (Fig. 2B). For this assay and all experiments going forward, the control strain was our COH1 dCas9 strain transformed with the knockdown plasmid carrying a “scramble” sgRNA (scrmbl), which is non-homologous to the COH1 genome, lacking any target.

Next, to verify the possibility of generating knockdowns of essential genes, a noted strength of CRISPRi, an sgRNA was produced to target the essential gene *ccpA*. After the transformation of this sgRNA into GBS, a growth curve was performed to verify a change in growth kinetics, and the difference was further quantified using an area under the curve (AUC) analysis. Again, we observed the expected phenotype, as the bacterial lag phase was extended by multiple hours (Fig. 2C), and area under the curve analysis showed a reduction in AUC (Fig. 2D). This further validates the CRISPRi system for reducing essential gene function without being lethal to the bacteria (32, 33). Taken together, the CRISPRi system produced targeted loss-of-function phenotypes as predicted.

### qPCR verification of transcriptional alteration

**Fig 3:**
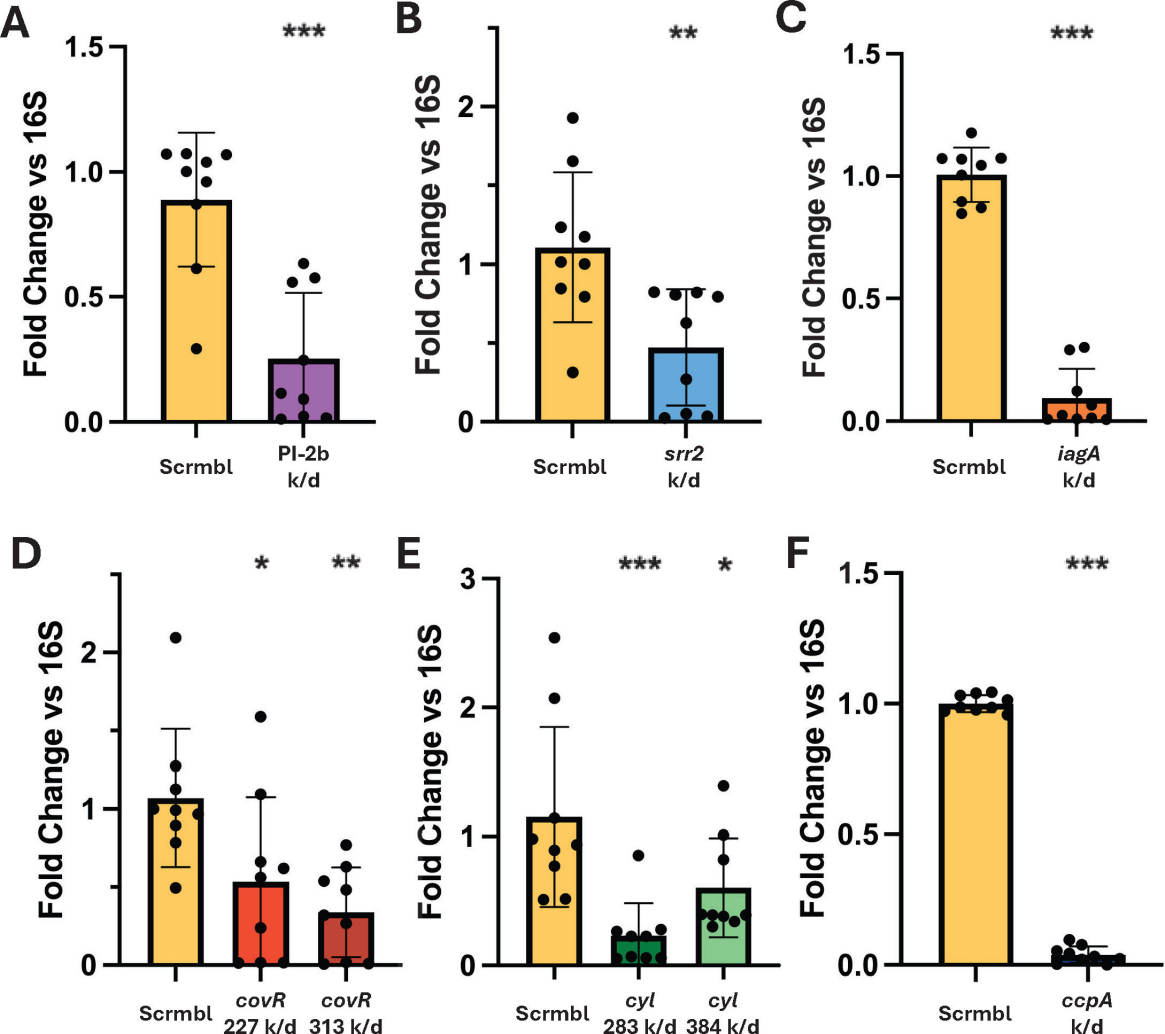
Transcriptional changes. (**A–F**) qPCR analysis of target genes following sgRNA transformation into *Streptococcus agalactiae* str. COH1 dCas9 for confirmation of CRISPR interference expression modulation. sgRNAs shown include the established virulence genes PI-2b (**A**), *srr2* (**B**), *iagA* (**C**), *covR* (**D**), and *cyl* (**E**), as well as the essential gene *ccpA* (**F**). (**A–F**) In each case, the control group is a COH1 dCas9 clone transformed with the “scramble” (scrmbl) sgRNA, which does not target the COH1 genome. All 16S normalization data are reported in Table S3. One-way ANOVA was performed, *P < 0.05, **P < 0.01, and ***P < 0.001. All experiments were performed in biological and technical triplicate (*n* = 9).

As CRISPRi reduces expression at the transcript level, qPCR was used to verify that the observed phenotypes were attributed to a reduction in targeted mRNA abundance. RNA was isolated via bead beating and standard lysis protocols, and cDNA was synthesized to be used in a qPCR reaction. Knockdown verification was performed using the previously mentioned genes *covR, cylE*, and *ccpA,* as well as in the established virulence factors *srr2*, *iagA*, and PI-2b. In each case, sgRNA-targeted genes showed reduced expression levels (Fig. 3), though some variability was apparent, with distance from transcriptional start not always trending with expression reduction. The primers used are reported in Table S1.

### CRISPRi for *in vitro* infection studies

**Fig 4:**
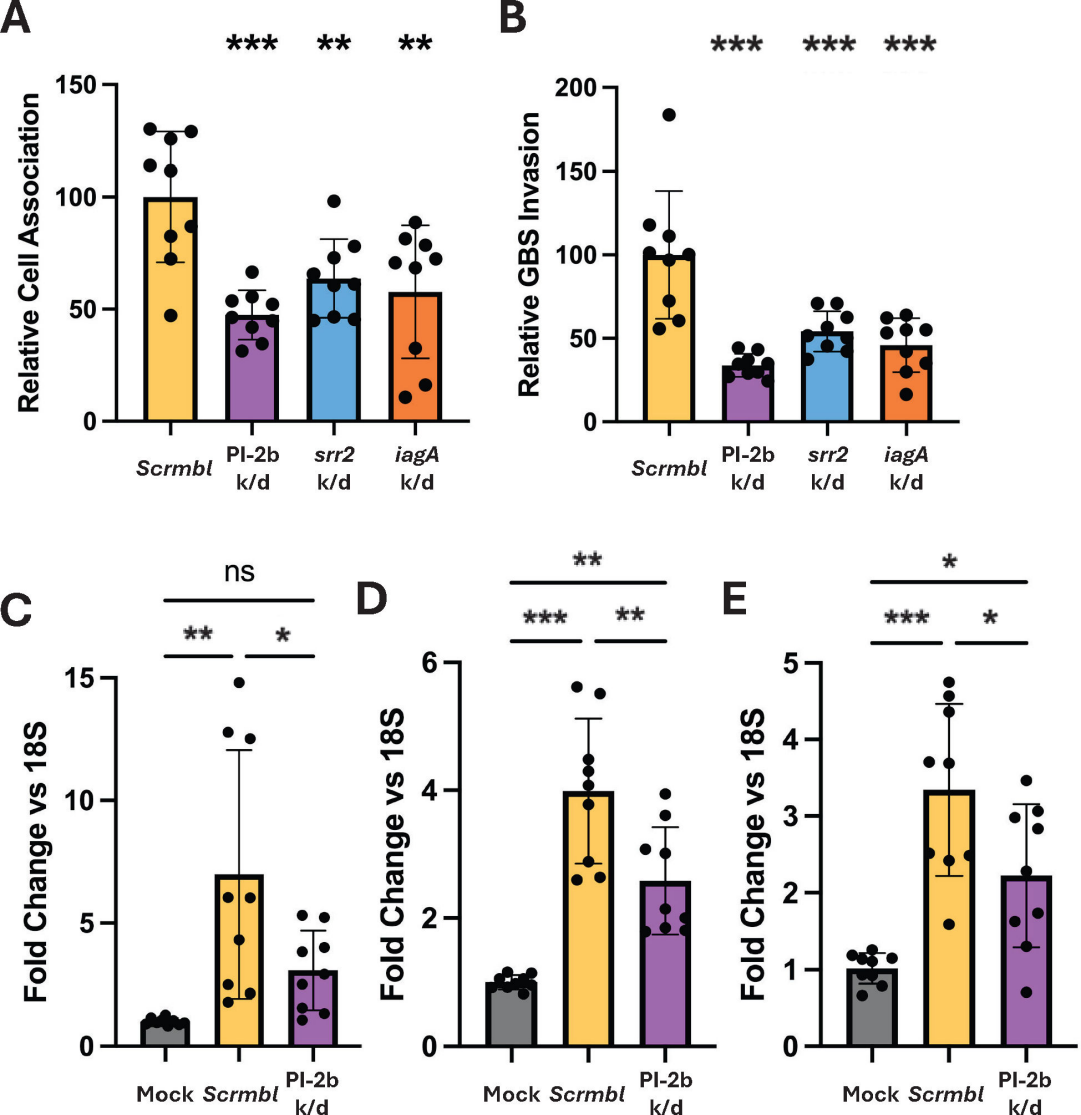
*In vitro* infection application. GBS adhesion (**A**) and invasion (**B**) rates to hCMEC/D3 monolayers when various well-established virulence genes are knocked down. (**C–E**) hCMEC/D3 inflammatory marker expression levels when facing challenge with either mock infection, “scramble” (scrmbl) control, or PI-2b knockdown as a representative virulence gene. Left to right, the qPCR targets are (**C**) *CXCL8* (IL-8), (**D**) *CXCL1*, and (**E**) *CXCL2*. All 18S normalization data are reported in Table S3. (**A–E**) One-way ANOVA was performed, *P < 0.05, **P < 0.01, and ***P < 0.001. All experiments were performed in biological and technical triplicate (*n* = 9).

As GBS COH1 is associated with neonatal meningitis, we sought to ensure that knockdowns of known factors in the CRISPRi strain would produce reproducible infection phenotypes in established *in vitro* blood-brain barrier modeling systems. For adhesion/invasion assays and cell immune response verification, the human brain endothelial cell line hCMEC/D3 was used. For investigating the efficacy of the CRISPRi model in infection, sgRNAs were designed to target the known GBS virulence factors PI-2b, s*rr2*, and i*agA*. These genes all have verified roles in infection at the BBB *in vitro* and with *in vivo* mouse models, as well as further investigation of the molecular mechanisms of these roles in infection (2, 14, 23, 24, 30). Adhesion (reported as cell-association) and invasion (bacterial cell internalization) results were quantified by infecting hCMEC/D3 cells seeded on a 12-well plate for 30 min and 2 h, respectively. All knockdowns exhibited a reduction in both adhesion (Fig. 4A) and invasion (Fig. 4B) relative to scramble. To investigate the known contribution of PI-2b to immune activation, we performed an experiment using mock-infected cells, infected with scramble, or infected with a PI-2b-targeted knockdown and collected RNA to quantify changes in chemokine and cytokine expression. We found that the scramble control significantly increased chemokine and cytokine expression when compared to mock, and that knocking down PI-2b reduced the overall induction of these proinflammatory genes (Fig. 4C through E). Taken together, these results demonstrate that use of the CRISPRi system mimics phenotypes previously described using classical mutagenesis, thereby providing an opportunity to use CRISPRi to identify potential novel targets in the future.

### *In silico* GBS CRISPRi sgRNA library generation

Following the generation of the CRISPRi strain and verification of its utility, we compiled an *in silico* high-coverage sgRNA library for use in GBS infection research using CRISPRi. We have designed a double-coverage library of potential sgRNAs using the software tool CHOPCHOP, covering 1,944/2,073 GBS genes (omitting rRNA and tRNA genes), totaling 3,595 sgRNA sequences (Table S2) to be used as a resource for GBS research. Each of these guides has been generated to minimize off-target effects and consistency of knockdown effects and evaluated for homology against several other common GBS research strains, including A909, CNCTC 10/84, NEM316, BM110, and CJB111 (Table S2). This *in silico* library can facilitate future GBS phenotypic research by speeding the design of synthetic protospacer oligonucleotides and could potentially inform the development of a full-genome coverage knockdown library in GBS.

## DISCUSSION

CRISPRi is an increasingly popular technology for precise, tunable gene regulation, and it has been utilized across a wide range of studies in both eukaryotic and prokaryotic systems. By using a catalytically inactive Cas9 (dCas9) targeted to specific genomic sequences via single-guide RNAs, CRISPRi enables researchers to inhibit transcription without permanently altering the genome, making it a powerful tool for investigating gene function (32, 33, 36, 39). CRISPRi applications in GBS are advancing, with new tools for GBS rapidly being developed (32, 36). These innovations create opportunities to advance the study of clinically relevant strains, such as COH1, as well as the study of complex host–pathogen interactions.

Our primary goal in designing this mutant-based endogenous CRISPRi system was ease of use, leveraging tools established in other GBS strains while balancing the knockdown efficiency. To that point, every guide tested in this study produced a viable knockdown phenotype, and none of the virulence knockdowns demonstrated an unexpected growth phenotype (Fig. S2). Notably, these phenotypes were typically achieved within 2–3 weeks from guide design to assay, depending on transformation success.

As expected, variability in knockdown efficiency was observed across different sgRNAs. This is consistent with prior reports demonstrating that repression efficiency is influenced by the distance of the guide from the transcriptional start site (TSS). For instance, the *cyl* guide targeted 283 bp from TSS showed a greater degree of knockdown than the guide targeted 384 bp from TSS (Fig. 2B), in agreement with an established correlation (33). The *covR* guide pair seems to disagree with this; however, more factors may be in play as the less effective *covR* 227 guide targets an AGG PAM sequence, which has been reported elsewhere as being a less efficient PAM sequence in GBS (32). Furthermore, we demonstrated successful repression of the essential gene ccpA, illustrating the potential of this system to interrogate genes for which deletion mutants cannot be generated (Fig. 2C and D).

Also of importance is the viability of the dCas9 mutant as a tool for the study of COH1 infection modeling *in vitro*. We and others utilize several models to this end, with the iBEC and hCMEC/D3 models increasing the robustness of the findings (31, 40, 41). Three well-established virulence genes were selected for knockdown: PI-2b*, iagA*, and *srr2*, as there is a great deal of literature to which we can compare our knockdown data for verification purposes. The data presented here compares as predicted to knockout phenotypes, though to a lesser extent in some cases (Fig. 4A and B) (2, 14, 23, 30). Additionally, we performed qPCR and found that CMEC/D3 cells had a reduced inflammatory response to knockdown strains of virulence factors, with neutrophil recruitment markers (*IL-8*, *CXCL1*, and *CXCL2*) expressed at lower levels after infection with the PI-2b knockdown strain compared to the control (Fig. 4C through E).

While the system shows strong potential utility, some limitations are worth noting, particularly given the use of a mutated endogenous Cas9 gene rather than purely plasmid-based systems of CRISPRi. Namely, antibiotic selection is necessary to retain the plasmid, which is a limitation for *in vivo* applications. It is also possible that there are unexpected non-transcriptional effects from mutating endogenous *Cas9* rather than a plasmid system. Regardless, this is unlikely as previous literature has shown minimal transcriptional change in GBS dCas9 strains relative to wild type, as PAM scanning is unaffected by this mutation (32). We also note one unexpected observation: the *ccpA* knockdown exhibited a longer lag phase yet ultimately reached a higher OD₆₀₀ than the scramble control. However, this atypical OD₆₀₀ profile likely reflects altered cell physiology rather than true differences in growth or arrangement, representing a minor interpretive limitation of optical-density-based measurements in this system as previously reported (32, 42). The generation of the *in silico* sgRNA library may be of use to other researchers studying GBS gene function, facilitating faster screening of genes and providing key validation before investing the time and resources to generate mutants. Future innovation on these systems may permit more complex studies, such as CRISPRi-seq, permitting the discovery and interrogation of new genes of interest in GBS COH1.

## MATERIALS AND METHODS

### Maintenance and differentiation of cell lines

hCMEC/D3 cells were cultured as described previously (43). Cells were maintained on tissue culture flasks coated with 1% rat tail collagen in EndoGRO MV medium (Millipore-Sigma). Cells were grown until 85% confluent, then split for infection experiments. Cells were seeded onto 12-well tissue culture plastic dishes coated with 1% rat tail at 1 × 10^5^ cells/cm^2^ for experiments and allowed to grow to confluency (4 days) at 37°C + 5% CO_2_ before infection experiments were conducted.

### Bacterial strains and growth conditions

Group B Streptococcus (GBS; *Streptococcus agalactiae*) strain COH1 (serotype III, multi-locus sequence type 17) was used for this study and cultured in Todd-Hewitt broth (THB) at 37°C in static culture (44, 45). *Escherichia coli* strain DH5α competent cells were used as a reservoir for plasmids and were grown in Luria-Bertani (LB) broth (LB Miller formulation) at 37°C.

### sgRNA design and protospacer cloning

Guides are designed as described previously to maximize efficiency (33, 39). Great care was taken during design to avoid off-target activity, avoiding seed-sequence homology wherever possible. With that in mind, the software tool CHOPCHOP was used for sgRNA design and NCBI BLAST was used extensively to verify sgRNA efficacy. Guides for *cyl* and *covR* were received from Dr. Thomas Hooven’s lab at the University of Pittsburgh Medical Center, as was the sgRNA shuttle vector p3015b (32). sgRNA sequences are ordered from Eurofins as individual oligos with desired overhangs matching the vector p3015b insertion site, then end phosphorylated (New England Biolabs T4 polynucleotide kinase). Phosphorylated oligos are annealed by heating to 95°C for 5 min, followed by gradual cooling to 4°C to produce the desired spacer. p3015b is miniprepped from DH5α *E. coli* before digestion with BsaI (Eco31I) (Thermofisher). The digested plasmid sample is cleaned (Promega Wizard SV Gel and PCR Cleanup Kit), then the annealed spacer is ligated into the digested shuttle vector (New England Biolabs T4 Ligase) for heat-shock transformation into *E. coli*. Following *E. coli* transformation, the plasmid is miniprepped (ThermoFisher PureLink Quick Plasmid Miniprep Kit) for transformation into GBS.

### *In silico* GBS sgRNA library design

In general, guides were designed as described above. For library generation, wherever possible, two sgRNAs were generated for every gene (omitting rRNAs and tRNAs) in the COH1 genome. For each gene, one sgRNA was designed near the TSS (−100 bp to 300 bp from TSS) and another further downstream (>500 bp downstream of TSS). Wherever possible, AGG PAM sequences were avoided due to the noted reduced efficiency of this PAM in GBS (32). The *silico* tool CHOPCHOP was used for sgRNA generation, as it is designed to help minimize off-target effects. Following sgRNA sequence generation, the best sequences were then checked for homology to other common GBS lab strains (A909, CNCTC 10/84, NEM316, BM110, CJB111), and sgRNAs that would be useful in other strains were chosen wherever possible. This library is provided in Table S2.

### GBS competent cell preparation and transformation

Electrocompetent GBS were prepared as described previously, with minor adaptations (46–48). COH1 was grown overnight in a 15 mL THB + 0.6% glycine broth at 37°C. The following day, this culture was diluted in an additional 35 mL of THB + 0.6% glycine and grown to OD_600_ = 0.6. This culture was pelleted via 4°C centrifugation at 3,200 × *g* and washed twice on ice with a cold 0.6% glycine solution. The remaining pellet was then resuspended in a 400 µL solution of cold 25% PEG 6000 + 10% glycerol and used immediately. Electroporation was also performed as described previously, with only minor alterations (46–48). When electroporating the bacteria, three 3 kV pulses were performed with 5 s intervals between them. Bacteria were then permitted to recover for 2 h in THB + 25% PEG 6000 in static culture before plating on THB + 5 µg/mL erythromycin agar plates.

### Generation of GBS dCas9 mutations

Point mutations needed for catalytic deadening of Cas9 were performed in two steps due to the size of the gene and the genomic distance between the two catalytic sites. The H845A missense mutation was generated as previously described using allelic exchange mutagenesis (48). The sucrose-sensitive suicide vector pMBSacB, containing the necessary homology repair cassette with the point mutation, was generously provided by Dr. Thomas Hooven’s lab at the University of Pittsburgh Medical Center. This plasmid was transformed into GBS as described above, and after screening via colony PCR, the transformants were grown overnight in THB + 5 ug/mL erythromycin broths at 28°C. The following day, these broths were passed to fresh tubes of the same broth at 37°C to begin temperature selection of the first allelic crossover. Following PCR verification of the first allelic crossover event, broths of this strain were passed into broths of THB lacking erythromycin to eliminate selection and permit curing of the plasmid. These broths were repeatedly passed at 28°C and plated onto THB until colony PCR, using primers 750 base pairs up-and-downstream of the homology arm sites, could confirm that a second allelic crossover event occurred. The product of this colony PCR was then inserted into a TOPO TA Vector (TOPO TA Cloning Kit for Sequencing, Thermofisher) and, after being passed in DH5α *E. coli*, submitted to Plasmidsaurus for Oxford Nanopore Sequencing to confirm the generation of the first point mutation.

The second point mutation was generated using the Cas12a expressing vector pGBSedit, which was received from Dr. Thomas Hooven’s lab at the University of Pittsburgh Medical Center. A 23 bp protospacer targeted to the Cas9 gene was designed using the appropriate TTTV PAM. This was assembled into pGBSedit (NEB HiFi Assembly 2x Master Mix), and then the homology repair cassette harboring the D10A point mutation was designed and also assembled into the vector. In order to prevent self-cutting of either the plasmid or the final, mutated genome by Cas12, the repair cassette also carries a series of silent mutations in the TTTV PAM used for targeting, as well as the targeted sequence itself. The remainder of the process was performed as previously described by Hillebrand et al. (36). The plasmid is electroporated into GBS, and, after screening using primers oJC270 and oJC271 and re-streaking, a colony is inoculated into THB + 5 µg/mL erythromycin broth and grown overnight. This broth is then sub-cultured, and eventually, 250 ng/mL anhydrotetracycline is added, followed by a 1-h incubation period. The culture is then plated onto THB agar + 5 ug/mL erythromycin + 250 ng/mL anhydrotetracycline agar plates and allowed to grow overnight. Mutant PCR screening for point mutation is performed by designing a primer (D10A Screen F) to match the silent mutated sgRNA targeting sequence of the mutant gene as the forward primer and using a primer 750 base pairs outside the homology region as the reverse (Cas9 PCR Out R). PCR was performed using the Monserate Midas Quik-Load PCR Mix 2X (2004-G). To ensure specificity for the point mutation, it is necessary to avoid using any polymerase mixes that contain isostabilizing compounds. Raising the annealing temperature 3°C–5°C above the norm for Taq PCR may also be necessary. The PCR-confirmed mutant was then re-streaked, cured of the plasmid by passes in media without erythromycin, and then the genome was sent to Plasmidsaurus for whole-genome sequencing to confirm the desired genotype. All primers are reported in Table S1.

### GBS infection assays

hCMEC/D3 or iBEC cells were seeded onto 12-well plates. Before infection assays, GBS *dcas*9 knockdown strains were grown overnight at 37°C in THB, supplemented with 5 µg/mL erythromycin to aid in selection for the p3015b vector containing the protospacer. From the overnight culture, bacteria were subcultured into fresh THB + 5 µg/mL erythromycin and grown to OD_600_ = 0.4–0.6. Bacteria were spun down and resuspended in PBS to an OD_600_ = 0.4. Bacteria were then diluted 1:10 in EndoGRO-MV Complete Culture Media Kit to a multiplicity of infection (MOI) of 10. Adherence and invasion assays were conducted following previously described protocols (49, 50).

For the bacterial adherence assays, bacteria and cells were incubated for 30 min at 37°C + 5% CO_2_. The cells were then washed 5× with PBS to remove non-adherent bacteria, lysed with 0.025% Triton X-100, diluted in PBS, and plated onto THB + 5 µg/mL erythromycin plates. Plates were incubated overnight at 37°C, with colonies being quantified the following day. For the invasion assays, bacteria and cells were incubated for 2 more hours before adding 100 µg/mL gentamicin to kill any non-intracellular bacteria, enabling the quantification of only the intracellular, invaded population. After 2 more hours of incubation at 37°C + 5% CO_2_, cells were washed 3× with PBS to remove antibiotics and plated onto THB plates + 5 µg/mL erythromycin plates. Plates were incubated overnight as above for quantification. Cell association and invasion were quantified with the formula $\frac{\left(\#CFU*dilution\;correction*volume\;correction\right)\;}{\left(Input\;CFU/Well\right)\;},$ and normalized to MOI association/invasion rates. The true MOI was determined for each experiment.

### Hemolysis assays

Sheep RBCs were prepared by washing 5 mL of defibrinated blood (Hemostat Labs) with an equal volume of Hank’s Balanced Salt Solution (HBSS) (VWR) at 4°C. The RBCs were pelleted by centrifugation at 500 × *g* for 15 min at 4°C and resuspended in 5 mL of HBSS. This washing step was repeated three times. A 0.1% Triton X-100 in PBS solution was prepared, if not previously made, to serve as a positive control. The washed RBCs were then pelleted by centrifugation at 500 × *g* for 15 min at 4°C. The RBC pellet (50 μL) was diluted into 5 mL HBSS to generate a 1% red blood cell suspension (RBCS).

A 96-well plate was used to set up the assay. Each well contained 100 μL of the 1% RBCS combined with 100 μL of the bacterial PBS suspensions or control solutions. The plate was incubated at 37°C with 5% CO_2_ for 1 h. Following incubation, the plate was centrifuged at 2,000 rpm for 5 min at 4°C. Supernatants (100 μL) were transferred to new wells on the same 96-well plate, and hemoglobin release was measured by recording absorbance at 415 nm using a spectrophotometer. Percent hemolysis was calculated relative to a positive control consisting of RBCs treated with 0.1% Triton X-100 (100 μL), which represented 100% hemolysis.

### RNA isolation and qPCR

When evaluating human cell inflammatory responses to knockdowns, hCMEC/D3 or iBEC cells were seeded onto 12-well plates and infected with GBS at an MOI of 10. Immediately after 5 h of infection, the total RNA was collected using the Macherey–Nagel NucleoSpin RNA kit (Macherey–Nagel). cDNA was synthesized with the qScript cDNA Synthesis kit (Quantabio), followed by SYBR Green (PowerUp SYBR Green Master Mix, Thermo Fisher) qPCR for each of the targets: IL-8, CXCL1, CXCL2, primers all listed in Table S1. 18S was used as the housekeeping gene for human cell lines.

For bacterial knockdown qPCR verification, bacteria were grown as described for infection experiments to an OD_600_ = 0.4 to ensure mid-log growth phase. Bacteria were resuspended in RA3 lysis buffer, then bead-beaten to lyse before being processed as described above. qPCR data were collected on the QuantStudio3 system (Applied Biosystems), and data are presented as fold change using the delta–delta–CT calculation (51). All primers are listed in Table S1. All 16S and 18S raw CT values have been reported in a new Table S3.

## Data Availability

The sgRNA library has been made available as Table S2.

## SUPPLEMENTAL MATERIAL

Figure S1 jb.00376-25-s0001.tif COH1 dCas9 strain growth kinetics.

Figure S2 jb.00376-25-s0002.tif Knockdown strain growth kinetics.

Supplemental legends jb.00376-25-s0003.docx Legends for Fig. S1 and S2, and Tables S1 to S3.

Table S1 jb.00376-25-s0004.xlsx Primers used.

Table S2 jb.00376-25-s0005.xlsx sgRNA library.

Table S3 jb.00376-25-s0006.xlsx Housekeeping qPCR values.

ASM does not own the copyrights to Supplemental Material that may be linked to, or accessed through, an article. The authors have granted ASM a non-exclusive, world-wide license to publish the Supplemental Material files. Please contact the corresponding author directly for reuse.
