# Supplementary figures and images for: CRISPR interference in a Streptococcus agalactiae multi-locus sequence type 17 strain
Source: J Bacteriol. 2026 Jan 14;208(2):e00376-25. doi: 10.1128/jb.00376-25 (PMC12918731; doi:10.1128/jb.00376-25)

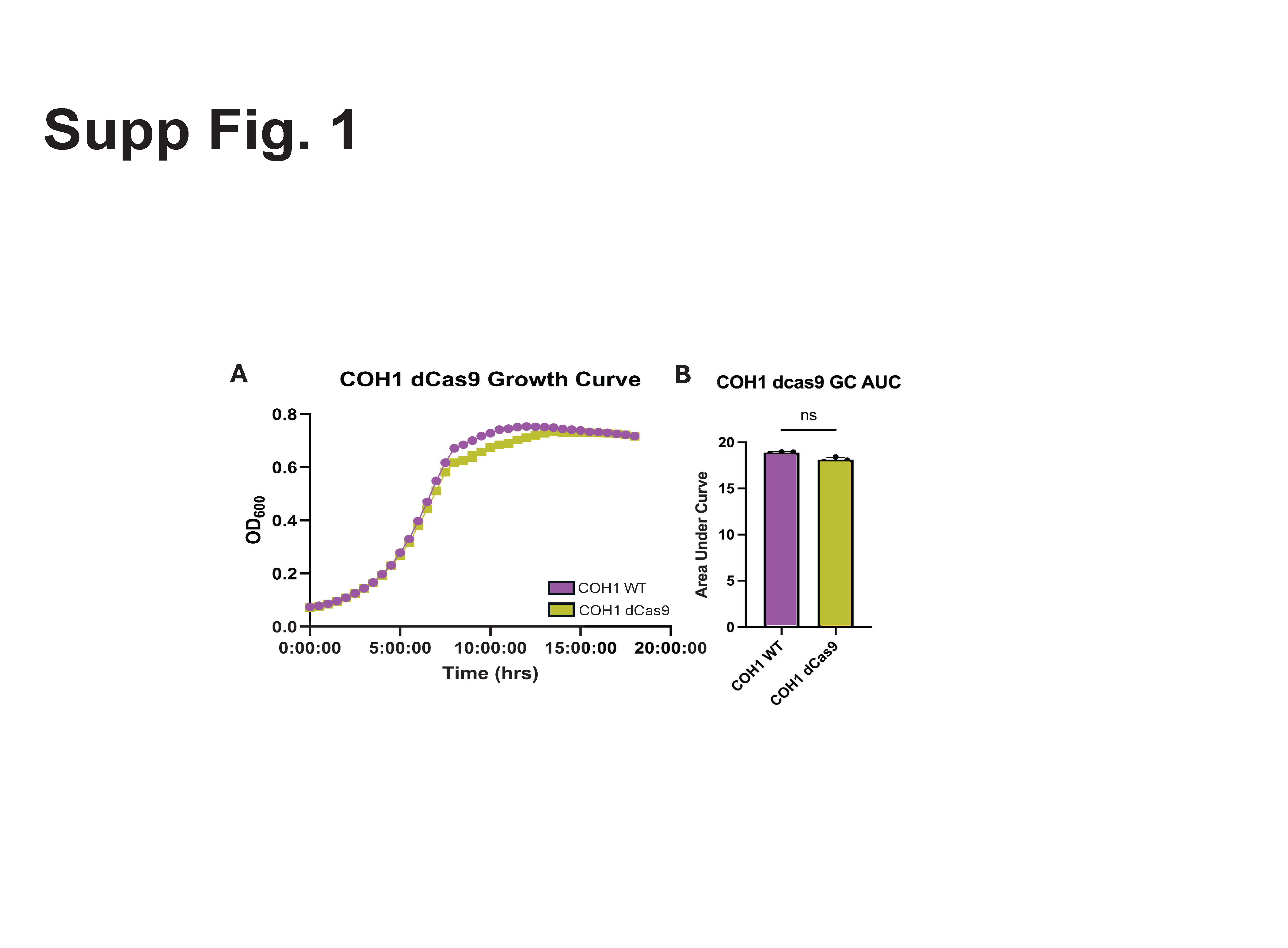

Supplement: Figure S1 — COH1 dCas9 strain growth kinetics. [file jb.00376-25-s0001.tif]

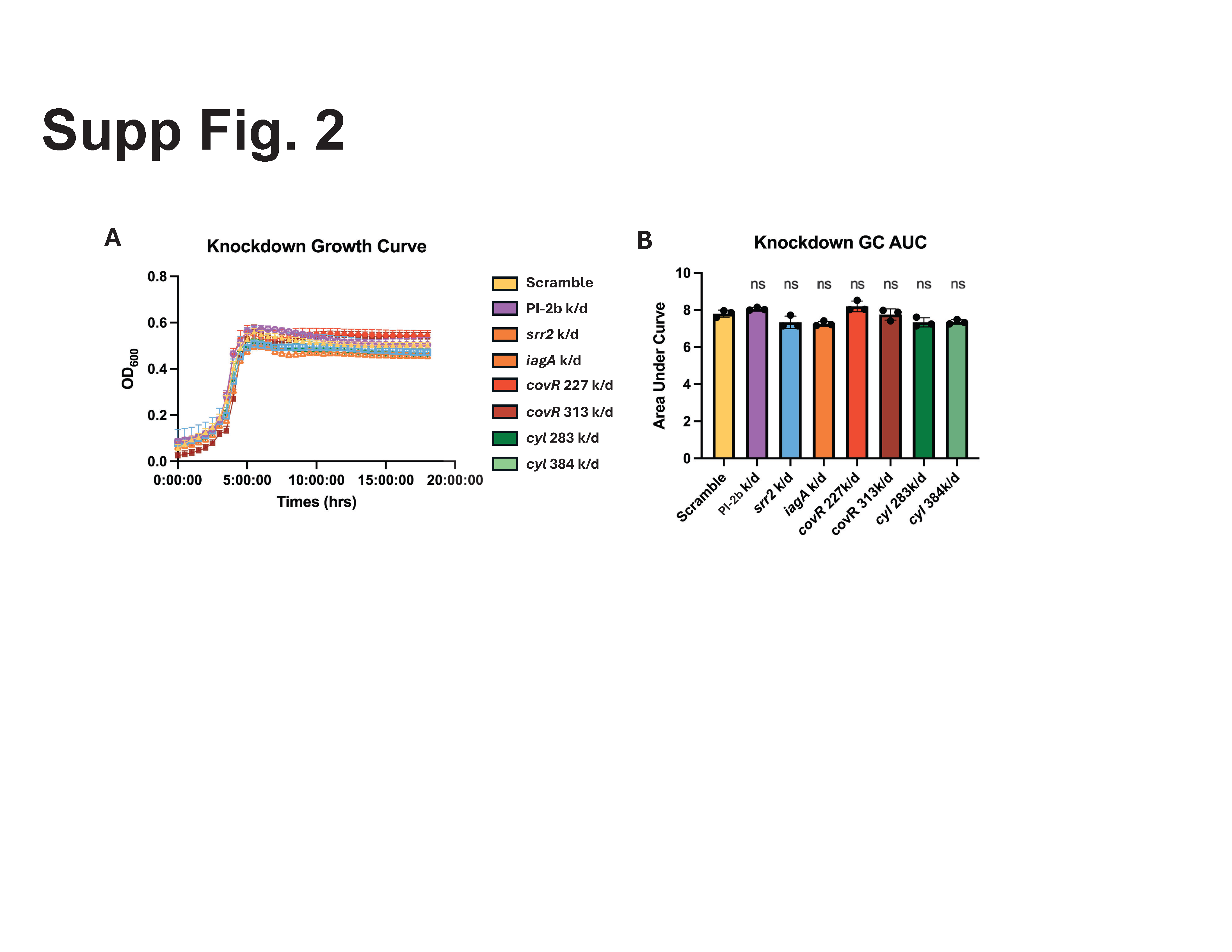

Supplement: Figure S2 — Knockdown strain growth kinetics. [file jb.00376-25-s0002.tif]
