# Supplementary material for: CRISPR interference in a Streptococcus agalactiae multi-locus sequence type 17 strain
Source: J Bacteriol. 2026 Jan 14;208(2):e00376-25. doi: 10.1128/jb.00376-25 (PMC12918731; doi:10.1128/jb.00376-25)
Supplement: Supplemental legends — Legends for Fig. S1 and S2, and Tables S1 to S3. [file jb.00376-25-s0003.docx]

**Supplemental Figure 1, COH1 dCas9 Strain Growth Kinetics.** (A) Growth kinetics curve showing the OD_600_ readings of the COH1 dCas9 mutant strain versus wild-type COH1. There is no observable difference, and (B) area under the curve analysis was performed using the biological replicates, with no significant change in area under the curve. The control for growth kinetics evaluation of the untransformed mutant strain is the wild-type COH1 strain. Student’s t-test was performed, nsP < 0.05. All experiments were performed in biological and technical triplicate.

**Supplemental Figure 2, Knockdown Strain Growth Kinetics.** (A) Growth kinetics curve showing the OD_600_ readings of the COH1 dCas9 mutant strain carrying the “scramble” sgRNA control versus the knockdowns sgRNAs targeted to PI-2b, *srr2*, *iagA*, *covR* 227, *covR* 313, *cyl* 283, *cyl* 384. There is no observable difference, and (B) area under the curve analysis was performed using the biological replicates, with no significant change in area under the curve among any of the gene knockdown groups relative to “scramble”. All significance reported represents comparisons to Scramble. One-way ANOVA was performed, nsP < 0.05. All experiments were performed in biological and technical triplicate.

**Supplemental Table 1, Primers Used.** Primers used in this publication for the purpose of mutagenesis or screening, as well as the “scramble” control sgRNA sequences used to interrogate knockdowns, along with descriptions of the use for each.

**Supplemental Table 2, sgRNA Library.** A double-coverage library of potential sgRNAs using the software tool CHOPCHOP, covering 1944/2073 GBS genes (omitting rRNA and tRNA genes), totaling 3595 sgRNA sequences. CHOPCHOP was used for sgRNA generation to help minimize off-target effects. Sequences were checked for homology to other common GBS lab strains (A909, CNCTC 10/84, NEM316, BM110, CJB111), and sgRNAs that would be useful in other strains were chosen wherever possible. The viable strains for each sgRNA are reported.

**Supplemental Table 3, Housekeeping qPCR Values.** Raw CT values reported that were used for qPCR normalization in other assays, sorted by replicate and group.
